# Palaeogenomics of the Hydrocarbon Producing Microalga *Botryococcus braunii*

**DOI:** 10.1038/s41598-018-38236-5

**Published:** 2019-02-11

**Authors:** Richard K. Tennant, Thomas M. Lux, Christine M. Sambles, Nikolaus J. Kuhn, Ellen L. Petticrew, Richard Oldfield, David A. Parker, Jackie Hatton, Karen A. Moore, Rob Lee, Chris S. M. Turney, Richard T. Jones, John Love

**Affiliations:** 1 Biosciences, College of Environmental and Life Sciences, The University of Exeter, Exeter, EX4 4QD UK; 2 Plant Genome and Systems Biology, Helmholtz Zentrum München, German Research Center for Environmental Health (GmbH), Munich, Germany; 3 Physical Geography and Environmental Change Research Group, University of Basel, Klingelbergstrasse 27, CH-4056 Basel, Switzerland; 4 Geography Program and Quesnel River Research Centre, University of Northern British Columbia, 3333 University Way, Prince George, BC V2N 4Z9 Canada; 5 Shell Technology Centre, 3333 Highway 6 South, Houston, Texas 77082-3101 USA; 6 Geography, College of Environmental and Life Sciences, The University of Exeter, Exeter, EX4 4RJ UK; 7 Palaeontology, Geobiology and Earth Archives Research Centre, School of Biological, Earth and Environmental Sciences, University of New South Wales, Sydney, NSW 2052 Australia

## Abstract

*Botryococcus braunii* is a colonial microalga that appears early in the fossil record and is a sensitive proxy of environmental and hydroclimatic conditions. Palaeozoic *Botryococcus* fossils which contribute up to 90% of oil shales and approximately 1% of crude oil, co-localise with diagnostic geolipids from the degradation of source-signature hydrocarbons. However more recent Holocene sediments demonstrate no such association. Consequently, *Botryococcus* are identified in younger sediments by morphology alone, where potential misclassifications could lead to inaccurate paleoenvironmental reconstructions. Here we show that a combination of flow cytometry and ancient DNA (aDNA) sequencing can unambiguously identify *Botryococcus* microfossils in Holocene sediments with hitherto unparalleled accuracy and rapidity. The application of aDNA sequencing to microfossils offers a far-reaching opportunity for understanding environmental change in the recent geological record. When allied with other high-resolution palaeoenvironmental information such as aDNA sequencing of humans and megafauna, aDNA from microfossils may allow a deeper and more precise understanding of past environments, ecologies and migrations.

## Introduction

*Botryococcus braunii* (*Trebouxiophyceae*; Chlorophyta)^1,2^ is colonial microalga found in fresh and brackish waters around the world^3–5^. *B. braunii* characteristically synthesise and secrete long-chain (C_18_–C_40_), liquid hydrocarbons, collectively termed botryococcenes^6–8^. *Botryococcus* appear early in the fossil record and are found globally in oil shales^9–15^ dating from the Precambrian (>542 Myr^16^), where they are the single largest biological contributor to crude oil^17^. In oil-shales, the dehydrogenated form of botryococcenes, termed botryococcanes^18^, are geolipids that co-localise with the fossil *Botryococcus*. *Botryococcus*-like microfossils are also prevalent in Holocene lacustrine sediments (younger than 11,650 cal yrs BP^19^), where they are used as essential proxies for reconstructing environmental and hydrological changes^20^ with high levels of temporal resolution^21^. In Holocene-age deposits however, *Botryococcus* sub-fossils are identified based on morphology alone which may result in inaccurate paleoenvironmental reconstructions.

The purification and analysis of ancient DNA (aDNA) from preserved materials has recently transformed our understanding of the phylogenies and migration patterns of extinct megafauna^22–24^ and humans^25,26^. Microfossils are much more common in sediment cores and widely used as proxies of environmental change but have not yet been subject to targeted genomic analysis. The application of aDNA sequencing to selected microfossils, in this case *Botryococcus*, offers considerable potential for enhanced analysis of the Holocene record. Moreover, the unambiguous identification of genetic material from *Botryococcus* fossils within Holocene-age sediments will allow detailed examination of the phylogenetic relationship between extinct and extant *Botyrococcus*, potentially providing insights to understand why *Botryococcus* synthesise and excrete their characteristic long-chain hydrocarbons^6–8^.

Here we investigate the potential for purifying *Botyrococcus* microfossils and aDNA from sediments known to contain the microalga. Conventional palaeoenvironmental analysis was used to determine the composition of the sediment in combination with palynological techniques to identify and quantify the putative *B. braunii* microfossils throughout a sediment core extracted from Boswell Lake, British Columbia, Canada. We first performed a two-dimensional gas chromatography analysis of the hydrocarbons present in the sediment to verify that the *Botryococcus* microfossils identified using these conventional techniques are co-localised with their associated source signature geolipids, as seen in oil shales. However, no such co-localisation was observed due to the migration and degradation of the diagnostic *Botryococcus* derived geolipids^27^. As a result, a key identifier of preserved *Botryococcus* in the Holocene record is unreliable and alternative corroboration is required.

We therefore used flow cytometry (FC) to rapidly distinguish, sort and collect the putative *Botryococcus* microfossils from defined sediment horizons, as previously performed for pollen grains^28^ and diatom frustules^29^. Preserved DNA was extracted from this material and sequenced using next generation Illumina sequencing. Sequences were aligned against a reference *B. braunii* genome^30^, providing unequivocal, molecular evidence of the identity of the microfossils. The combination of FC purification and DNA sequencing has wider applications to other microfossil species and the interpretation of their fossil record.

## Site Selection

**Figure 1:**
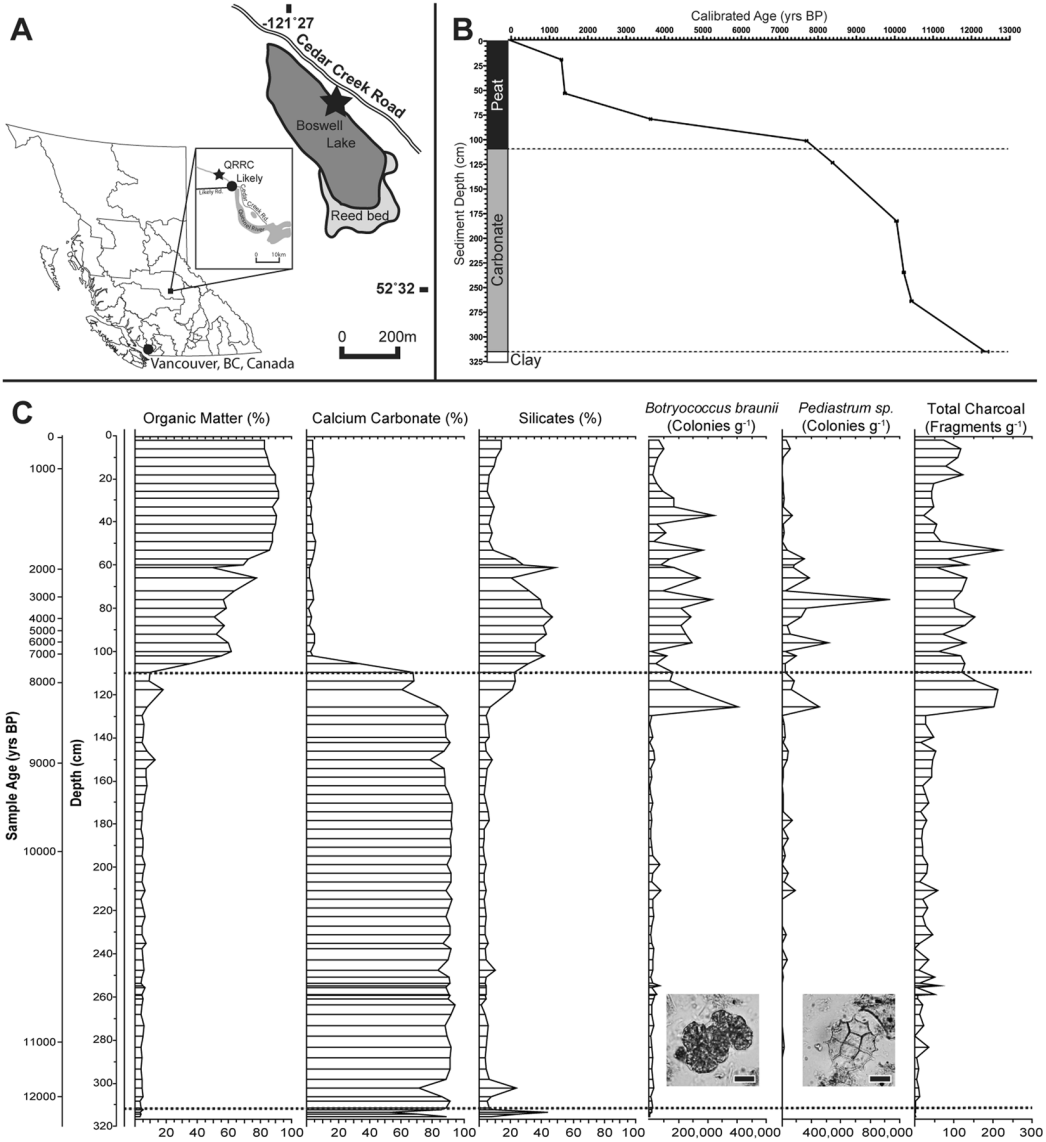
Boswell Lake Location and Sediment Analysis. (**A**) Map of British Columbia, Canada, highlighting Vancouver, and zoomed insert of Likely, Quesnel River Research Centre (QRRC) and surrounding area. Boswell Lake shown in dark grey with reed bed towards south east aspect highlighted in lighter grey. (**B**) Calibrated ages of sediment plotted against the sediment depth from which they originated. Sediment composition shown and lines indicate the depths at which sediment composition changes. (**C**) Summary of LOI analysis, charcoal, *Botryococcus braunii* and *Pediastrum sp*. concentration, visually identified in Boswell Lake sediment. Inset images of *B. braunii* and *Pediastrum sp.* which were identified. Lines indicate changes in sediment composition. Scale bars represent 20 µm.

A complete Holocene sediment record was recovered from Boswell Lake (Fig. 1), a carbonate lake located in British Columbia, Canada (52°32′24.72″N 121°27′5.23″W). Boswell Lake is situated in the Cariboo region of the interior of British Columbia and neighbours Quesnel Lake, the third deepest lake in North America^31^. The site was selected because *Botryococcus* had previously been morphologically identified throughout the sediment record at Boswell Lake, predominantly from 8,400 cal yrs BP. While pristine environments are arguably rare on Earth^32^, Boswell Lake is extremely remote with minimal human influence on the lake or sediments, making it an ideal site for aDNA analysis.

## Hydrocarbons

**Figure 2:**
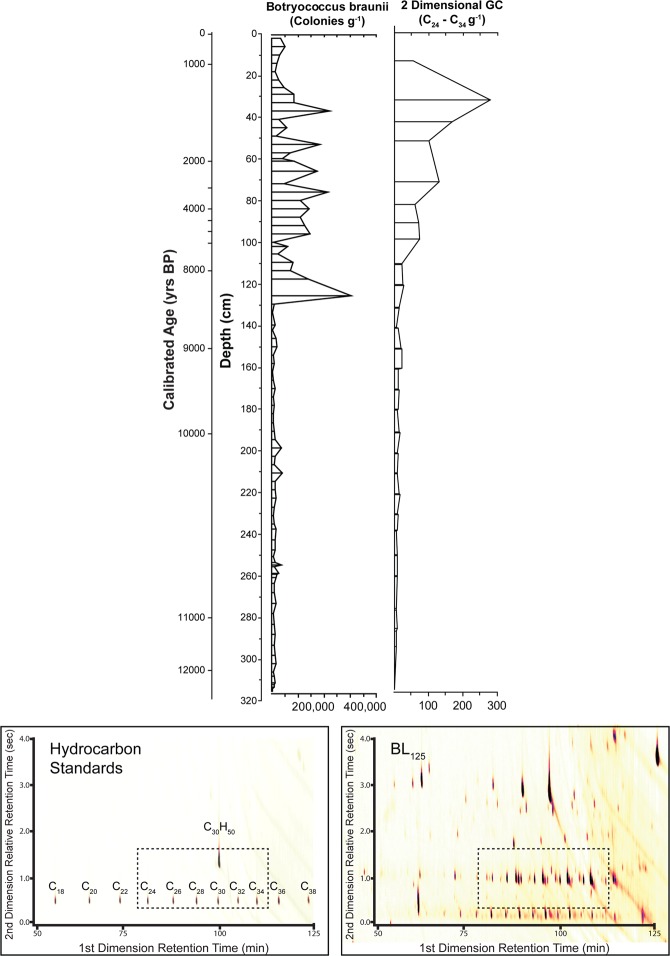
Comparison of *Botryococcus braunii* identified by Microscopy and Chemical Biomarkers. Upper left: *B. braunii* concentration, visually identified in Boswell Lake sediment. Upper right: Summary of C_24_–C_34_ hydrocarbons concentration analysed be 2-dimensional (2D) gas chromatography. Lower left: 2-D gas chromatograph indicating the retention times of an C_18_–C_38_ alkane ladder and squalene standard. Lower right: 2D gas chromatograph of representative sample BL_125_. A region of interest, indicated by the black box, was used to quantify the *B. braunii* type hydrocarbons.

The total concentration of the C_24_–C_34_ hydrocarbons produced by *B. braunii* were normalised to one gramme of sediment from each horizon and used to generate a hydrocarbon profile of the entire sediment core (Fig. 2).

The concentration of C_24_–C_34_ hydrocarbons measured in defined horizons through the sediment core of Boswell Lake (Fig. 2) did not correlate to the concentration of *B. braunii* colonies visually identified within the matching sediment but show a rapid decrease in concentration after the top metre of the core. The geolipids therefore do not correlate with the counts of visually identified *B. braunii*, which could be attributed to the compression of sediments over time^27^. This confirms that while geolipids may be used to identify the presence of specific organisms or taxonomic groupings within Holocene sediments generally, potential upwards migration and microbial degradation of hydrocarbons suggests that these biomarkers cannot be used to emphatically prove that the visually identified algal subfossils at a specific horizon are indeed *B. braunii*.

## Purification of putative *B. braunii* colonies using flow cytometry

**Figure 3:**
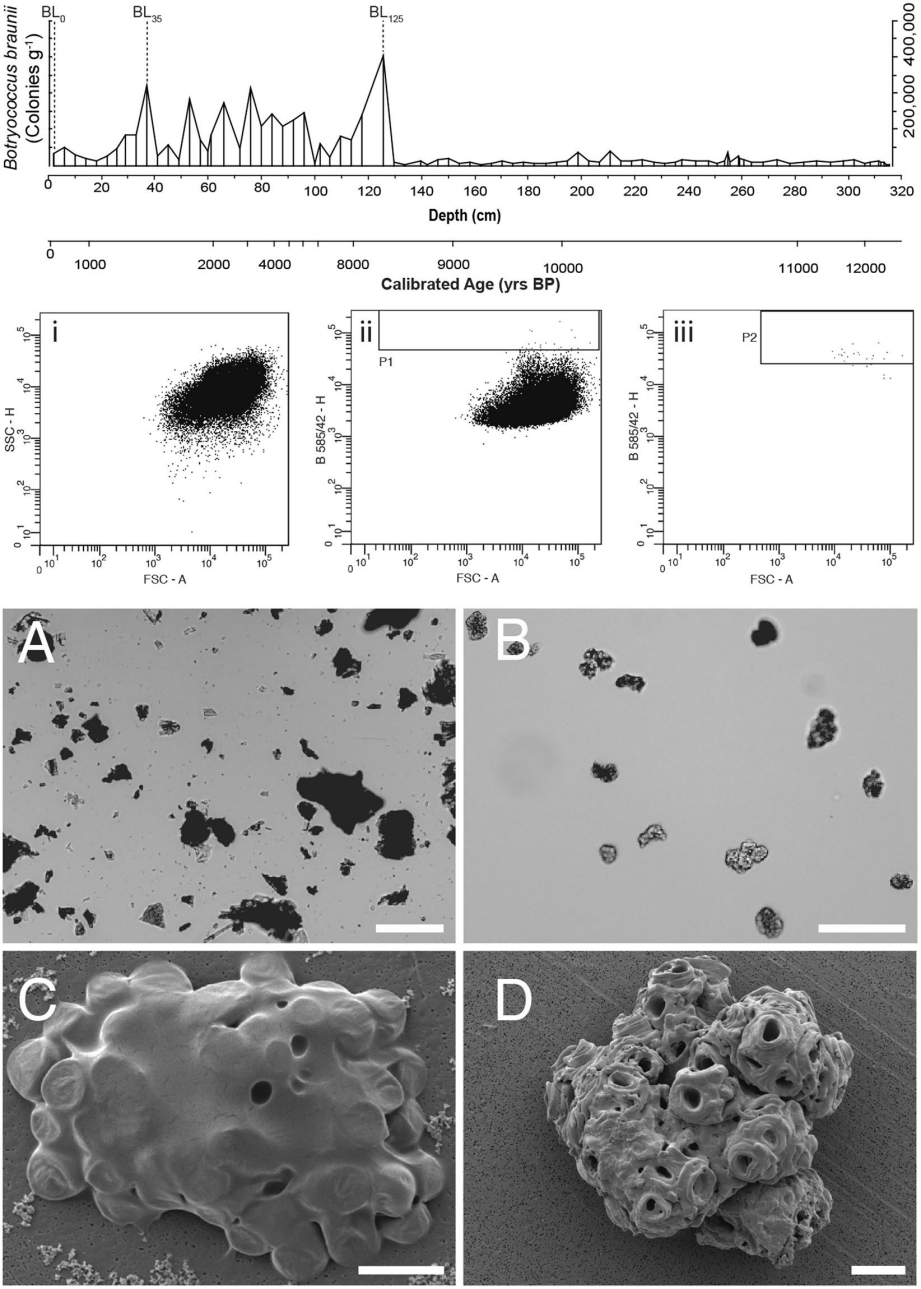
Flow Cytometry Dot Plots and Images. Top - *Botryococcus braunii* concentration, visually identified in Boswell Lake sediment. i – Forward scatter *vs*. side scatter height, an indicator of the scatter of Boswell Lake sediment particles. ii – Forward scatter *vs*. 488 nm excitation, 585/42 nm emission height measurement, particles that are fluorescing intensely gated as P1. iii - Forward scatter *vs*. 405 nm excitation, 450/40 nm emission height measurement, particles from P1 displayed, those which exhibit high fluorescence are gated P2. The P2 is used to sort *B. braunii* from Boswell Lake sediment. (**A**) Bright field image of prepared Boswell Lake sediment before analysis by flow cytometry. (**B**) Bright field image of sorted P2 region from Boswell Lake sediment. (**C**) SEM image of extant *B. braunii* colony, covered in hydrocarbon sheath. Associated bacteria can also be seen. (**D**) SEM image of assumed *B. braunii* sorted from Boswell Lake sediment using flow cytometry. For (**A**,**B**) the scale bar represents 100 µm, for (**C**,**D**) the scale bar represents 10 µm.

Flow cytometry was used to sort 10,000 putative *Botrycoccus* colonies (Fig. 3) from three horizons at, 0 cm (BL_0_), 35 cm (BL_35_) and 125 cm (BL_125_) from the surface. Morphology of the sorted microfossils were verified by light (Fig. 3A,B) and scanning electron microscopy (Fig. 3C,D) and compared to those of the modern culture of *B. braunii* culture and published images of fossil *Botryococcus* (Fig. 3)^33^. The sorted samples from Boswell Lake were morphologically similar to the images of extinct *Botroycoccus* and extant *B. braunii*. However, the characteristic geolipid signature and observed microfossils were not present in the same strata as they are in more ancient sediments, therefore calling into question the precise nature of these microfossils. In addition to the microfossils sorted BL_0_, BL_35_ and BL_125_ sediment samples, control samples comprising 10,000 *Pinus* pollen grains from the same horizon as BL_0_, 10,000 and 100,000 10 µm calibration beads, were sorted using flow cytometry, to identify any nucleic acid contamination from the sorting procedure.

## Phylogenetic Assessment

**Figure 4:**
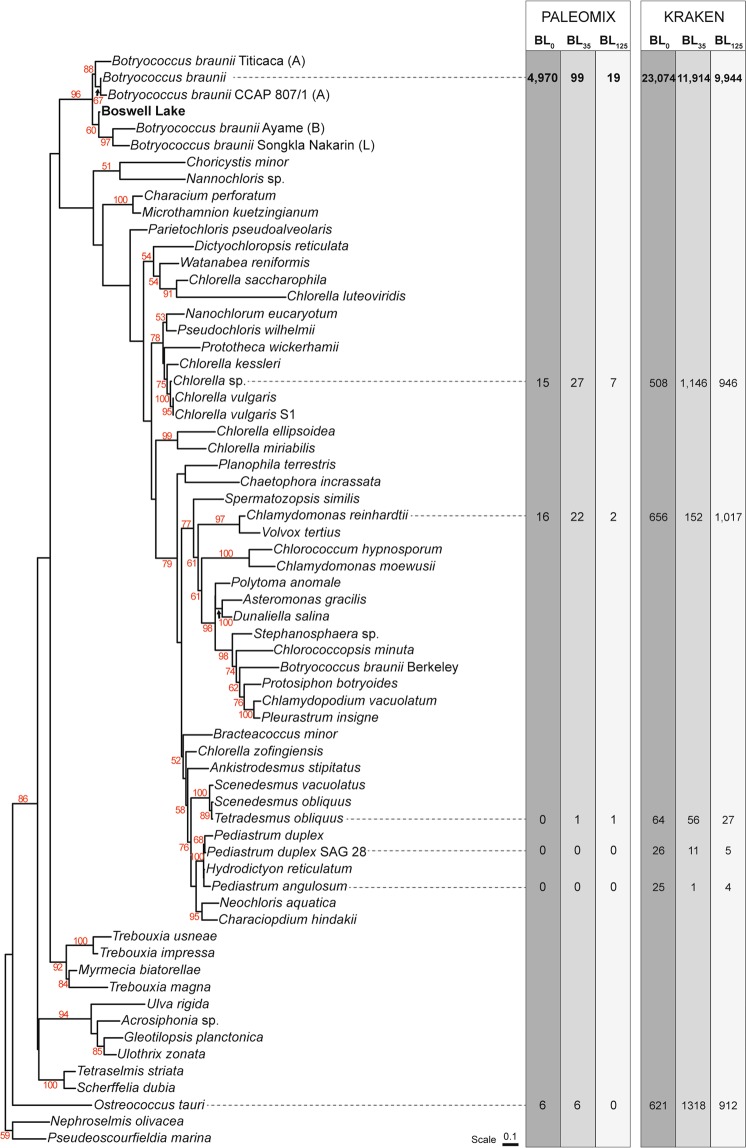
Phylogenetic Assessment of DNA Extracted from FACS-Purified, Subfossil *Botryococcus braunii*. Phylogenetic tree of the 18S rRNA gene coding sequences from Boswell Lake *B. braunii* and algal taxa generated using PhyML. The bootstrap percentage values are displayed in red for branches that have credibility values above 50%. The number of DNA sequence reads from samples BL_0_, BL_35_ and BL_125_ which align to these taxa using PALEOMIX and Kraken2 is displayed.

To determine the relationship between the *B. braunii* identified in the recently incorporated (BL_0_) Boswell Lake sediment, extant *B. braunii* and a proven set of selected algal species^2^, a phylogenetic classification was performed using the 18S rRNA gene coding sequence (Fig. 4). The phylogenetic comparison assigned the sequences from the BL_0_ horizon to the same branch as that of extant *B. braunii* and supports the likelihood that the *B. braunii* identified in the Boswell Lake sediment is indeed *B. braunii*, and not another colonial microalga. Although DNA was purified and sequenced from BL_35_ and BL_125_, 18S rRNA gene sequences could not be identified in the DNA sequence reads from these horizons, potentially due to the sediments’ age and DNA degradation. Consequently, we analysed further the aDNA sequence reads from purified microfossils from BL_0_, BL_35_ and BL_125_ respective to the draft *B. braunii* genome.

## Ancient DNA Analysis

While the morphology of the putative subfossil *Botryococcus* is comparable to that of extant *B. braunii*, in the absence of a reliable geolipid profile, molecular verification is necessary to confirm the genus unequivocally. This is the first time a subfossil microalga has been characterised using aDNA at genomic level. DNA was purified from each of the FACS sorted and control samples, DNA sequencing libraries were prepared and sequenced using an Illumina MiSeq (Supplementary Table 1). DNA sequence reads were quality assured before alignment to the genome of *B. braunii*^30^ using PALEOMIX^34^, ensuring complete matches between the DNA sequence reads and the reference genome (Table 1). For BL_0_ 4,970 reads aligned to the *B. braunii* Showa reference genome, 99 reads from BL_35_ aligned and 19 reads aligned from sample BL_125_. For the controls, 4 reads aligned to the *B. braunii* reference from the 10,000 bead control and no reads aligned from the 100,000 FACS sorted bead controls, while 5 reads aligned from the pollen control sample (Table 1).

**Table 1:** Alignment of DNA sequence reads extracted from FACS purified subfossil *Botryococcus braunii* to the nuclear and organelle genomes of extant *B. braunii*.

Sample	Core Horizon (cm)	Age (cal yr. BP)	Sequence Reads Aligned to *B. braunii*		
			Nuclear genome	Mitochondria	Chloroplast
BL_0_	0	0	4,970	18	5
BL_35_	35	1,350	99	0	0
BL_125_	125	8,400	19	0	0
*Pinus* Pollen	35	0	4	0	0
FACS Beads (10k)	—	—	0	0	0
FACS Beads (100k)	—	—	5	0	0s

The cytosine to thymine deamination of the 5′ and 3′ termini of the aligned reads was visualised using the mapDamage^35^ package within PALEOMIX (Supplementary Fig. 1). There is no deamination observed in BL_0_ which is expected for modern samples. The profiles for samples BL_35_ and BL_125_ display evidence of 5′ and 3′ cytosine to thymine deamination, indicating that the purified DNA originated from ancient samples. The DNA sequence reads from samples BL_0_, BL_35_ and BL_125_ were aligned using PALEOMIX to genomes of selected *Chlorophyta* taxa (*B. braunii* Showa., *Chlorella* sp., *Chlamydomonas reinhardtii, Tetradesmus obliquus*, *Pediastrum angulosum*, *Pediastrum duplex* and *Ostreococcus tauri*), to determine the validity of the assignments (Fig. 4). The maximum number of hits for BL_0_, BL_35_ and BL_125_ was against the *B. braunii* reference genome, while the number of alignments for BL_35_ and BL_125_ is inconclusive when compared to the number of alignments against other taxa this analysis is strongly suggestive that these algae microfossils are most likely *B. braunii*. The DNA sequence reads from BL_0_, BL_35_ and BL_125_ were also independently aligned to a custom-built database of the same *Chlorophyta* taxa using Kraken2^36^, which corroborated the analysis performed by PALEOMIX (Fig. 4); 23,074 reads aligned from BL_0_, 11,914 reads aligned from sample BL_35_ and 9.944 reads aligned from BL_125_ to the *B. braunii* reference genome. A comparative analysis was performed of the DNA sequence reads which aligned using PALEOMIX and those which aligned using Kraken2 (Supplementary Fig. 2). 4,392 DNA sequence reads from sample BL0 aligned to *B. braunii* using either PALEOMIX and Kraken2, while only 1 DNA sequence read shared alignment between PALEOMIX and Kraken2 for BL_35_ and 3 DNA sequences reads aligned to the *B. braunii* reference genome from BL_125_ when using PALEOMIX and Kraken2. Within the caveats of aDNA preservation and an incomplete, available genome for *Botryococcus* compared to other Chlorophytes, the data strongly suggest that the purified microfossils are most likely *B. braunii*.

In addition to the alignments to the nuclear genome, the DNA sequence reads were compared to the organelle chloroplast (NC_025545.1) and mitochondria (NC_027722.1) genomes of *B. braunii* as they are available in the NCBI reference database. The increased copy number per cell of these organelle genomes may result in a greater proportion of positive alignments. There were 18 reads from BL_0_ that aligned to the mitochondrion genome and 5 reads which aligned to the chloroplast genome. There were no reads which aligned to either organelle for samples BL_35_ and BL_125_ or any of the controls (Table 1).

The combination of morphological, chemical, genomic and phylogenetic analysis provides compelling evidence that the microfossils identified in Boswell Lake sediment are indeed *B. braunii*. However, more aDNA is required to confirm the phylogenetic assessment of the *B. braunii* microfossils at Boswell Lake, necessitating fresh sediment samples and a higher number of *B. braunii* microfossils to be purified.

Here we demonstrate for the first time that it is possible to obtain *B. braunii* microfossils for aDNA analysis from sediments dating back to at least 8,400 cal yrs BP. Conventional techniques were unable to obtain a single type of microfossil from lacustrine sediments in sufficient quantity and purity for aDNA sequencing. However, using a combination of two high throughput and sensitive techniques we extracted sufficient microfossils from the sediment to purify enough high-quality aDNA for next-generation sequencing analysis. This technological combination provides evidence that the morphologically identified *Botryococous* are correctly classified as *B. braunii*, even in the absence of congruent geolipid signatures. Samples of this age are subject to DNA degradation which may explain the decreasing number of DNA sequence reads that align to the modern reference genome (Fig. 4). An alternative interpretation is differences between the DNA sequence reads and the only available reference genome, *B. braunii* race B, Showa^30^, could account for the observed reduction in reads aligned to the reference genome for samples extracted from BL_35_ and BL_125_. Future genomic assembly of the four reported races of extant *B. braunii*^37–40^, in addition to a more robust and diverse reference database, would enable an insight into the success of *B. braunii* hydrocarbon synthesis and secretion over many millennia.

Our results provide supporting evidence for palaeoenvironmental reconstruction. For instance, following the last glacial period, summer temperatures in British Columbia reached a thermal maximum some 10,500 to 8,400 cal yrs BP^41–43^. Intriguingly, *B. braunii* is thought to prevail in equable environments, especially after a wet period^44^. The unequivocal classification of the microfossils as *B. braunii* therefore confirms that stratigraphy and enables confident paleoenvironmental analysis. Unfortunately, the DNA sequence data acquired in this study cannot be used to perform significant comparisons between the extant *B. braunii* genome and that of the microfossils which exceed simple identification of the species. However, as more data become available, understanding the molecular evolution of *B. braunii*, and the divergence of the four extant *B. braunii* races from a putative ancestor becomes a real possibility. Moreover, the combination of high throughput, purification of targeted microfossils and DNA sequencing developed here can be applied to other microalgal species and subfossils, such as pollen and spores, and enables unprecedented sensitivity for resolving and understanding the species and environments during periods of abrupt and extreme change which prevailed through the Quaternary^45–47^.

## Methods

### Core Extraction and Sub Sampling

A complete 3.20 m sequence of deposits were taken using a 5 cm diameter Livingstone corer to give minimal sediment disturbance^48^. Cores were recovered in 1 m sections and the corer cleaned between samples. Cores were sealed in the field and transported back to the Quesnel River Research Centre for processing. The sediment core was subsequently split into 1 cm contiguous subsamples for analysis.

### Core Reconstruction

Samples were prepared for multi-proxy analysis using the standard preparation method for sub-fossil extraction^49^. *B. Braunii*, *Pediastrum sp*. and charcoal concentrations were determined by the addition of tablets containing a known concentration of *Lycopodium* spores to each sample prior to counting and calculated following standard protocols^50^. Organic, Carbonate and Silicate content of the cores was analysed by loss-on-ignition^51,52^.

### Radiocarbon Dating

Pollen was concentrated from defined horizons of sediment by flow cytometry^28^. Plant and animal macrofossils were also dated from horizons which exhibited suitable samples. Samples were dated at either the University of Waikato or the University of Oxford Radiocarbon Accelerator Unit (ORAU) and dates calibrated using Northern Hemisphere IntCal13, which are expressed in calendar years Before Present (CE 1950)^53^. Supplementary Table 2.

### Sediment Hydrocarbon Analysis by Two-Dimensional Gas Chromatography

Hydrocarbons were extracted from freeze-dried sediment horizons using a Dionex ASE-200 (California, U.S.A.). A known mass of sediment was placed into an Accelerated Solvent Extractor (ASE) cartridge and processed using 10 ml of dichloromethane at 1500 psi and 150 °C. Solvent extracts were dried under argon to a volume between 1 and 2 ml. All dried extracts were corrected to volume of 2 ml using dichloromethane in a volumetric flask. Prepared samples were analysed using gas chromatography coupled with a flame ionisation detector (Agilent 7890 GC FID, California, U.S.A.). 1 µl of sample was splitless injected. Samples were also analysed of known standards and each of the modern *B. braunii* races A, B & L to confirm the region of the chromatograph where the Botryococcus derived branched C_24_–C_34_ hydrocarbons appear.

### Sediment Preparation for *Botryococcus* Microfossil Extraction

The conventional chemical preparation of sediment samples for the visual identification of subfossil material involves hydrochloric acid (HCl), hydrofluoric acid (HF), acetic acid (CH_3_COOH), sulphuric acid (H_2_SO_4_), sodium hydroxide (NaOH) and heating the sample to 95 °C. It was considered likely that the exposure of samples to this chemical preparation would have a detrimental effect on the quantity and quality of any DNA preserved within them. Therefore the following preparation method was devised which minimised both the chemical treatment and removed the necessity for heating the sample.

0.5 g of freeze dried sediment was added to a 50 ml centrifuge tube. Approximately 20 ml of 1 M HCl was slowly added until effervescence ceased. The suspension was left loosely capped for 15 min so any residual CO_2_ from the reaction could escape. Samples were centrifuged at 3,000 r.c.f. for 10 min. The supernatant was removed and 20 ml of sterile, milli-Q H_2_O was added to the pellet, which was re-suspended by vortex-mixing for 1 min. The centrifugation and wash process was repeated once more. Samples were passed through a 106 µm steel sieve and collected on a 10 µm nylon sieve cloth. The remaining 10 < 106 µm fraction was passed through a 100 µm nylon cell strainer to ensure all particles that were greater than 100 µm (*i.e*. larger than the nozzle of the flow cytometer) were removed. Samples were deflocculated in a sonicating water bath for 10 min to remove any aggregates.

### High-throughput Extraction of Microfossils from Sediments

Prepared samples were analysed by fluorescence using a BD FACS Aria II (Becton Dickenson, USA) equipped with a 100 µm nozzle. Particle forward scatter and side scatter were obtained using a 488 nm laser and the appropriate detectors. Particle fluorescence was excited at 405 nm and at 488 nm, and fluorescence intensity recorded at 530 ± 30 nm and at 585 ± 42 nm respectively. Samples were sorted into autoclaved glass 5 ml vials, which were capped and stored at 4 °C.

### Light and Scanning Electron Microscopy

Micrographs were acquired using either a Leica DCF300FX digital camera coupled to a Leica MZ16F dissecting microscope (436/20 nm excitation 480/40 nm emission, 470/40 nm excitation, 510 nm long pass emission and 525/50 nm emission, 10x zoom) and controlled with Leica FireCam Software or a Leica DM2500 compound microscope coupled with a Q-Imaging Micropublisher 3.3 camera, controlled with Q-Capture software. All prepared samples were imaged prior to analysis by flow cytometry. Selected sorted samples which were to be subjected to SEM imaging were placed onto a Nuclepore^TM^ polycarbonate filter and washed three times with 10 ml milli-Q H_2_O to remove any residue of PBS that would cause salt crystals to form on the Nuclepore^TM^ filter. The resultant filter paper was placed onto an SEM stub and sputter coated (Q150T-ES, Quorum Technologies, UK). The prepared sample was visualised by SEM (JSM-6390LV SEM, JEOL, Japan) and images captured.

### DNA Purification

DNA was purified using the FastDNA for Soil DNA Extraction Kit (MPBio, 116560200) following the manufacturer’s instructions with a final elution volume of 100 µl. All manipulations were performed inside a class II laminar flow cabinet. Purified DNA was quantified using the Qubit assay (Thermo-Fisher Scientific, Horsham, UK). The quality of the purified DNA was assured using either the 2100 Bioanalyzer (Agilent, U.S.A.) with High Sensitivity DNA Chips (Agilent, U.S.A.), following the manufacturer’s instructions or the D1000 Tapestation (Aglient, U.S.A.) with High Sensitivity ScreenTape (Agilent, U.S.A.) following the manufacturer’s instructions. Purified DNA was stored at −20 °C until further analysis was performed.

### Preparation of DNA Sequencing Library

Purified DNA was fragmented by using a Covaris E220 focused-ultrasonicator (Covaris, Massachusetts, U.S.A.), which was programmed for a target fragment size of 500 bp; 105 W, 5% duty factor, 200 cycles per burst, 80 s treatment time. Fragmented DNA was purified by bead purification using Agencourt AMPure XP beads (Beckman Coulter, California, U.S.A.). DNA sequencing libraries were prepared using the NuGen Ovation Ultralow DR kit (0330-32 NuGen, California, U.S.A.).

### DNA Sequencing

DNA sequencing of the prepared libraries was performed by the University of Exeter sequencing service, who sequenced the DNA libraries using an Illumina MiSeq, using either 250 bp paired end or 300 bp paired end sequencing.

### Bioinformatic Methods

Sequence data was accessed and all subsequent analysis was performed using a local server containing 32 3.1 GHz CPUs and 256 Gb RAM. The system was installed with Fedora v.21 Linux operating system.

### DNA Sequence Quality Control and Validation

DNA sequence data quality was analysed and visualised using FastQC^54^ http://www.bioinformatics.babraham.ac.uk/projects/fastqc/. DNA sequences reads which had a Phred score below 20 were removed using Trim-Galore^55^.

### Phylogenetic Assessment

Quality controlled and verified DNA sequence reads from BL_0_ were aligned to the 18S rRNA gene coding sequence from *B. braunii* Ayamé AJ581910 using bwa mem^56^. A consensus sequence was extracted using samtools^57^ and aligned against 18S rRNA gene coding sequences from a robust and published phylogeny of 57 different algal species^2^ in addition to *Ostreococcus tauri* GQ426346, *Chlorella vulgaris* KX618655, *Tetradesmus obliquus* KX618656, *Pediastrum angulosum* AY663032*, Pediastrum duplex* AY780662, *Volvox tertius* FJ610144 and *Botryococcus braunii* KR869723 using MUSCLE^58^. Maximum likelihood analysis was performed using PhyML 3.1^59^, a GTR substitution model with four substitution rates and a gamma shape parameter of 0.477. A total of 100 bootstrap replications were performed. The resultant tree was drawn using iToL v4^60^.

### Alignment to Published Genomes

DNA sequence reads were aligned against the genomes of *B. braunii* Showa., *Chlorella* sp., *Chlamydomonas reinhardtii, Tetradesmus obliquus*, *Pediastrum angulosum*, *Pediastrum duplex* and *Ostreococcus tauri* by PALEOMIX^34^ and a custom database containing these genomes using Kraken2^36^.

### Alignment to Organelle Genomes

Assembled scaffolds from each sediment horizon was aligned to the available genomes of the *B. braunii* chloroplast (NCBI: NC_025545) and mitochondrion (NCBI: NC_027722) using bwa mem^56^.

## Supplementary information

Palaeogenomics of the Hydrocarbon Producing Microalga <i>Botryococcus braunii</i>. Supplementary Information

## Data Availability

Raw data is deposited in the NCBI Sequence Read Archive under BioProject ID PRJNA474793. The phylogenetic alignment and tree have been submitted to TreeBASE, Study ID – 23659.

## References

[1] Kützing, F. T. *Species algarum*. 1–922 (F. A. Brockhaus, 1849).

[2] Senousy HH, Beakes GW, Hack E (2004). Phylogenetic placement of Botryococcus braunii (Trebouxiophyceae) and Botryococcus sudeticus isolate UTEX 2629 (Chlorophyceae). Journal of Phycology.

[3] Metzger, P., Largeau, C. & Casadevall, E. In *Fortschritte der Chemie organischer Naturstoffe/Progress in the Chemistry of Organic Natural Products* **57**, 1–70 (Springer Vienna, 1991).

[4] Wolf FR, Nonomura AM, Bassham JA (1985). Growth and Branched Hydrocarbon Production in a Strain of Botryococcus Braunii (Chlorophyta). Journal of Phycology.

[5] Metzger P, Largeau C (2005). Botryococcus braunii: a rich source for hydrocarbons and related ether lipids. Appl. Microbiol. Biotechnol..

[6] Maxwell JR, Douglas AG, Eglinton G, McCormick A (1968). The Botryococcenes—hydrocarbons of novel structure from the alga Botryococcus braunii. Kützing..

[7] Berkaloff C, Casadevall E, Largeau C, Peracca MS, Virlet J (1983). The resistant polymer of the walls of the hydrocarbon-rich alga Botryococcus braunii. Phytochemistry.

[8] Banerjee A, Sharma R, Chisti Y, Banerjee UC (2002). Botryococcus braunii: A Renewable Source of Hydrocarbons and Other Chemicals. Critical Reviews in Biotechnology.

[9] Volkman JK (2014). Acyclic isoprenoid biomarkers and evolution of biosynthetic pathways in green microalgae of the genus Botryococcus. Organic Geochemistry.

[10] Thiessen, R. Origin of the boghead coals. *Professional Paper* 121–137 (1925).

[11] Ji L-M, Yan K, Meng F-W, Zhao M (2010). The oleaginous Botryococcus from the Triassic Yanchang Formation in Ordos Basin, Northwestern China: Morphology and its paleoenvironmental significance. Journal of Asian Earth Sciences.

[12] Traverse A (1955). Occurrence of the Oil-Forming Alga Botryococcus in Lignites and Other Tertiary Sediments. Micropaleontology.

[13] Testa, M., Gerbaudo, S. & Andri, E. In *Proceedings of the Ocean Drilling Program, 180 Scientific Results* **180**, (Ocean Drilling Program, 2002).

[14] David, T. *Note on the origin of kerosene shale*. (Proc. Linnean Soc. NSW, 1889).

[15] Adam P, Schaeffer P, Albrecht P (2006). C40 monoaromatic lycopane derivatives as indicators of the contribution of the alga Botryococcus braunii race L to the organic matter of Messel oil shale (Eocene, Germany). Organic Geochemistry.

[16] Gradstein FM, Ogg JG, Smith AG, Bleeker W, Lourens LJ (2004). A new Geologic Time Scale, with special reference to Precambrian and Neogene. Episodes.

[17] Moldowan, J. M. & Seifert, W. K. First discovery of botryococcane in petroleum. *J. Chem. Soc., Chem. Commun*. 912–914, 10.1039/c39800000912 (1980).

[18] Hunt, J. M. *Petroleum geochemistry and geology*. (W.H. Freeman New York, 1979).

[19] Walker M (2009). Formal definition and dating of the GSSP (Global Stratotype Section and Point) for the base of the Holocene using the Greenland NGRIP ice core, and selected auxiliary records. J. Quaternary Sci..

[20] Rawson DS (1956). Algal Indicators of Trophic Lake Types. Limnology and Oceanography.

[21] Tang L (2013). Palaeoecological and palaeoenvironmental significance of some important spores and micro-algae in Quaternary deposits. Chinese Science Bulletin.

[22] Kosintsev P (2018). Evolution and extinction of the giant rhinoceros Elasmotherium sibiricum sheds light on late Quaternary megafaunal extinctions. Nature Ecology & Evolution.

[23] Metcalf JL (2016). Synergistic roles of climate warming and human occupation in Patagonian megafaunal extinctions during the Last Deglaciation. Science Advances.

[24] Cooper A (2015). Abrupt warming events drove Late Pleistocene Holarctic megafaunal turnover. Science.

[25] Librado P (2017). Ancient genomic changes associated with domestication of the horse. Science.

[26] Valdiosera C (2018). Four millennia of Iberian biomolecular prehistory illustrate the impact of prehistoric migrations at the far end of Eurasia. Proceedings of the National Academy of Sciences.

[27] Meyers PA (1997). Organic geochemical proxies of paleoceanographic, paleolimnologic, and paleoclimatic processes. Organic Geochemistry.

[28] Tennant RK (2013). A new flow cytometry method enabling rapid purification of fossil pollen from terrestrial sediments for AMS radiocarbon dating. J. Quaternary Sci..

[29] Tennant RK, Jones RT, Love J, Lee R (2013). A new flow cytometry method enabling rapid purification of diatoms from silica-rich lacustrine sediments. J Paleolimnol.

[30] Browne DR (2017). Draft Nuclear Genome Sequence of the Liquid Hydrocarbon-Accumulating Green Microalga Botryococcus braunii Race B (Showa). Genome Announc.

[31] Gilbert R, Desloges JR (2012). Late glacial and Holocene sedimentary environments of Quesnel Lake, British Columbia. Geomorphology.

[32] Boivin NL (2016). Ecological consequences of human niche construction: Examining long-term anthropogenic shaping of global species distributions. Proceedings of the National Academy of Sciences.

[33] Zippi PA (1998). Freshwater Algae from the Mattagami Formation (Albian), Ontario: Paleoecology, Botanical Affinities, and Systematic Taxonomy. Micropaleontology.

[34] Schubert M (2014). Characterization of ancient and modern genomes by SNP detection and phylogenomic and metagenomic analysis using PALEOMIX. Nat Protoc.

[35] Jónsson H, Ginolhac A, Schubert M, Johnson PLF, Orlando L (2013). mapDamage2.0: fast approximate Bayesian estimates of ancient DNA damage parameters. Bioinf..

[36] Wood DE, Salzberg SL (2014). Kraken: ultrafast metagenomic sequence classification using exact alignments. Gen. Biol..

[37] Metzger P, Templier J, Largeau C, Casadevall E (1986). An n-alkatriene and some n-alkadienes from the A race of the green alga Botryococcus braunii. Phytochemistry.

[38] Metzger P, Casadevall E, Pouet MJ, Pouet Y (1985). Structures of some botryococcenes: branched hydrocarbons from the b-race of the green alga Botryococcus braunii. Phytochemistry.

[39] Metzger, P. & Casadevall, E. Lycopadiene, a tetraterpenoid hydrocarbon from new strains of the green alga Botryococcus braunii. **28**, 3931–3934 (1987).

[40] Kawachi M, Tanoi T, Demura M, Kaya K, Watanabe MM (2012). Relationship between hydrocarbons and molecular phylogeny of Botryococcus braunii. Algal Research.

[41] Chase M, Bleskie C, Walker IR, Gavin DG, Hu FS (2008). Midge-inferred Holocene summer temperatures in Southeastern British Columbia, Canada. Palaeogeography, Palaeoclimatology, Palaeoecology.

[42] Barber DC (1999). Forcing of the cold event of 8,200 years ago by catastrophic drainage of Laurentide lakes. Nature.

[43] Turney CSM, Brown H (2007). Catastrophic early Holocene sea level rise, human migration and the Neolithic transition in Europe. Quaternary Science Reviews.

[44] Cane, R. F. In *Developments in Petroleum Science* **5**, 27–60 (Elsevier, 1976).

[45] Brook EJ, Buizert C (2018). Antarctic and global climate history viewed from ice cores. Nature.

[46] Turney CSM (2015). Obliquity‐driven expansion of North Atlantic sea ice during the last glacial. Geophysical Research Letters.

[47] Rasmussen SO (2014). A stratigraphic framework for abrupt climatic changes during the Last Glacial period based on three synchronized Greenland ice-core records: Refining and extending the INTIMATE event stratigraphy. Quaternary Science Reviews.

[48] Wright HE (1967). A square-rod piston sampler for lake sediments. Journal of Sedimentary Research.

[49] Fægri, K. & Iversen, J. *Textbook of pollen analysis*. (John Wiley & Sons Ltd., 1989).

[50] Stockmarr J (1971). Tablets with spores used in absolute pollen analysis. Pollen et Spores.

[51] Dean WE (1974). Determination of carbonate and organic matter in calcareous sediments and sedimentary rocks by loss on ignition; comparison with other methods. Journal of Sedimentary Research.

[52] Bengtsson, L. & Enell, M. In *Handbook of Holocene palaeoecology and palaeohydrology* (ed. Berglund, B. E.) 423–451 (1986).

[53] Reimer, P. J. *et al*. IntCal13 and Marine13 Radiocarbon Age Calibration Curves 0–50,000 Years cal BP. *Radiocarbon; Vol 55, No. 4 (2013) KW - 55*, 1869–1887 (2013).

[54] Andrews, S. FastQC - A quality control tool for high throughput sequence data. (2010).

[55] Kruegar, F. B Bioinformatics - Trim Galore! (2014).

[56] Li H, Durbin R (2010). Fast and accurate long-read alignment with Burrows-Wheeler transform. Bioinf..

[57] Li H (2009). The Sequence Alignment/Map format and SAMtools. Bioinf..

[58] Edgar RC (2004). MUSCLE: multiple sequence alignment with high accuracy and high throughput. Nuc. Aci. Res..

[59] Guindon S (2010). New algorithms and methods to estimate maximum-likelihood phylogenies: assessing the performance of PhyML 3.0. Syst. Biol..

[60] Letunic I, Bork P (2016). Interactive tree of life (iTOL)v3: an online tool for the display and annotation of phylogenetic and other trees. Nuc. Aci. Res..

