# Supplementary material for: Palaeogenomics of the Hydrocarbon Producing Microalga Botryococcus braunii
Source: Sci Rep. 2019 Feb 11;9:1776. doi: 10.1038/s41598-018-38236-5 (PMC6370823; doi:10.1038/s41598-018-38236-5)
Supplement: Supplementary file 1 — Palaeogenomics of the Hydrocarbon Producing Microalga Botryococcus braunii. Supplementary Information [file 41598_2018_38236_MOESM1_ESM.pdf]

Palaeogenomics of the Hydrocarbon Producing Microalga *Botryococcus braunii.*
Supplementary Information

Richard K. Tennant[1], Thomas M. Lux[2], Christine M. Sambles[1], Nikolaus J. Kuhn[3], Ellen L. Petticrew[4], Richard Oldfield[5], David A. Parker[1,5], Jackie Hatton[6], Karen A. Moore[1], Rob Lee[1], Chris S. M. Turney[7], Richard T, Jones[6] and John Love[1*].

[1] Biosciences, College of Environmental and Life Sciences, The University of Exeter, Exeter, EX4 4QD, UK

[2] Plant Genome and Systems Biology, Helmholtz Zentrum München, German Research Center for Environmental Health (GmbH), Munich, Germany

[3] Physical Geography and Environmental Change Research Group, University of Basel, Klingelbergstrasse 27, CH-4056 Basel, Switzerland

[4] Geography Program and Quesnel River Research Centre, University of Northern British Columbia, 3333 University Way, Prince George BC Canada V2N 4Z9

[5] Shell Global Solutions, Shell Technology Centre, 3333 Highway 6 South, Houston, Texas, 77082-3101, USA

[6] Geography, College of Environmental and Life Sciences, The University of Exeter, Exeter, EX4 4RJ, UK

[7] Palaeontology, Geobiology and Earth Archives Research Centre, School of Biological, Earth and Environmental Sciences, University of New South Wales, Sydney, NSW 2052, Australia.

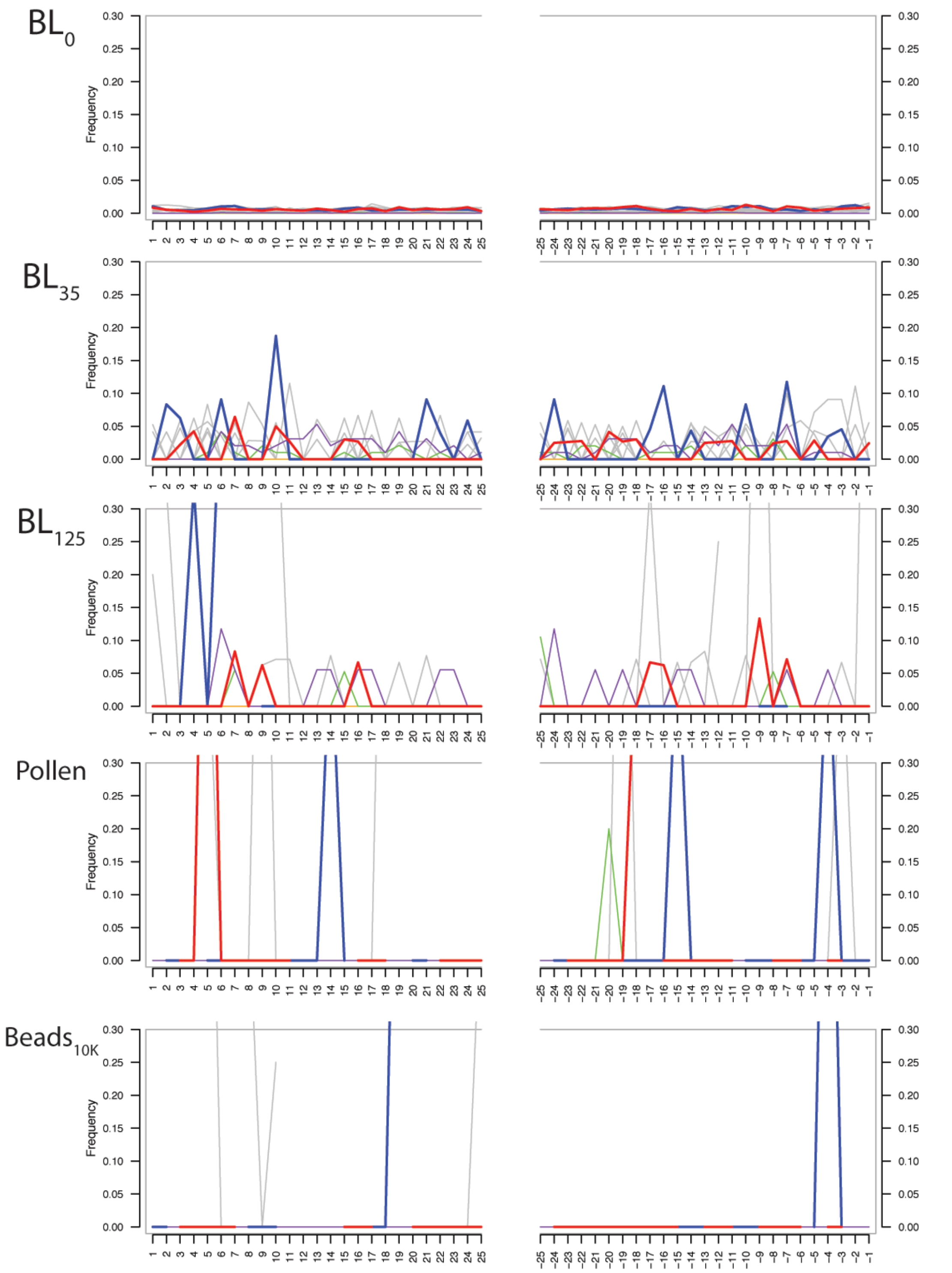

Supplementary Figure 1. MapDamage mis-incorporation plots of samples which aligned to the *B. braunii* reference genome.

MapDamage mis-incorporation plots for samples BL_0 BL_{35} BL_{125}, Pollen and 10K beads against the *Botryococcus braunii* Showa reference genome. Left panel represents the 5' DNA terminus and right panel represents the 3' DNA terminus. C to T substitutions shown in red; G to A substitutions shown in blue; all other substitutions shown in grey. Deletions are shown in green and insertions shown in purple. No data is shown for the 100,000 bead controls as no DNA sequence reads aligned.

Sample	Depth (cm)	Age (cal yr BP)	Purified DNA (ng/ml)	Average Size (bp)	Prepared Library Average Size (bp)	Prepared Library Concentration (nmol l-1)	Unique DNA Sequences
BL_0	0	0	25	14,578	656	180.6	10,212,204
BL_{35}	35	1,352	146	9,444	451	51.7	20,741,232
BL_{125}	125	8,406	26.8	7,890	501	92.6	14,601,912
Pollen	-	-	0	-	-	-	554,890
10K Beads	-	-	0	-	-	-	197,194
100K Beads	-	-	0	-	-	-	96,530

Supplementary Table 1. Samples of *Botryococcus braunii* sorted by flow cytometry from Boswell Lake and concentration of DNA purified from 10,000 events. '-' indicates below detectable limits.

Depth (cm)	Material Dated	14C age ± 1SD BP	Median Age (cal yr BP)	Laboratory Reference
20	Pollen	1,405 ± 25	1,312	Wk-36776
54	Plant Macrofossil	1,519 ± 30	1,402	ORAU OxA-26238
80	Pollen	3,392 ± 25	3,636	Wk-36777
102	Pollen	6,874 ± 27	7,700	Wk-36778
124	Pollen	7,554 ± 27	8,378	Wk-41331
183	Shell	8,930 ± 38	10,046	ORAU P-31600
235	Pollen	9,088 ± 33	10,237	Wk-41332
264	Pollen	9,257 ± 35	10,439	Wk-41333
315	Plant Macrofossil	10,460 ± 90	12,357	ORAU OxA-26239

Supplementary Table 2. Plant and animal macrofossils dated from defined horizons. Samples were dated at either the University of Waikato or the University of Oxford Radiocarbon Accelerator Unit (ORAU).

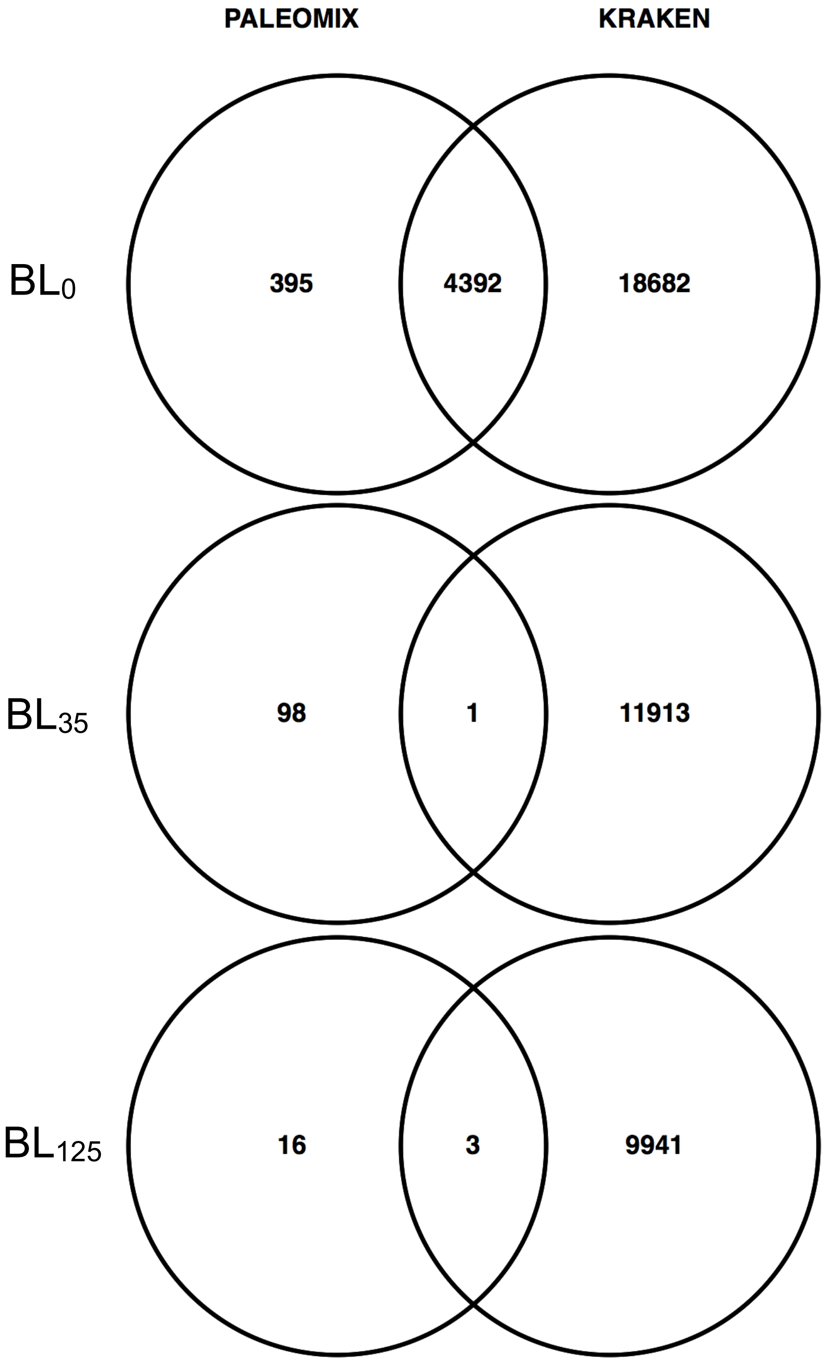

Supplementary Figure 2. Venn Diagrams displaying the overlapping DNA sequence reads of samples which aligned to the *B. braunii* reference genome using PALEOMIX and Kraken2

Venn diagrams of the number of DNA sequence reads from samples BL_0, BL_{35} and BL_{125} that align to *B. braunii* using either PALEOMIX, Kraken2 or both. Venn diagrams produced using Venny 2.1
